# Adolescent Risky Alcohol Use Is Associated With Electrophysiological Markers of Error Processing: Findings From a Large Cohort Study

**DOI:** 10.1016/j.bpsgos.2025.100615

**Published:** 2025-09-17

**Authors:** Olga D. Boer, Hanan El Marroun, Doga Ultanir, Ingmar H.A. Franken

**Affiliations:** a Center for Substance Use and Addiction Research, Department of Psychology, Education and Child Studies, Erasmus School of Social and Behavioral Science, Erasmus University Rotterdam, Rotterdam, the Netherlands; b The Generation R Study Group, Erasmus MC University Medical Center Rotterdam, Rotterdam, the Netherlands; c Department of Child and Adolescent Psychiatry, University Medical Center Rotterdam, Erasmus MC, Sophia Children’s Hospital, Rotterdam, the Netherlands

**Keywords:** Adolescence, Alcohol use, EEG, Error processing, ERP, Time-frequency

## Abstract

**Background:**

Altered electrophysiological error processing, measured via the error-related negativity (ERN), error-related positivity (Pe), and midfrontal theta power (FM-theta), has been associated with problematic alcohol use and alcohol dependence in clinical populations. However, large-scale studies focusing on adolescent use in the general population are scarce. Moreover, the extent to which potential confounding factors shape the relationship between brain activity and alcohol use remains unclear.

**Methods:**

In the current study, we examined the relationship between adolescent alcohol use and electrophysiological markers of error processing (ERN, Pe, and FM-theta) in a large adolescent sample drawn from a population-based cohort (*N* = 1525, 806 female, mean age = 18.4). Alcohol use variables included initiation, age at initiation, recent alcohol use quantity, and recent binge drinking frequency. Confounders included sex, IQ, socioeconomic factors, and alcohol-related risk factors such as prenatal alcohol and tobacco exposure or a parental history of substance use disorder.

**Results:**

Linear regression analyses showed that smaller absolute ERN amplitude (indicating reduced implicit error processing) was associated with risk factors for alcohol use disorder, including early alcohol use initiation and higher binge drinking frequency. Surprisingly, higher binge drinking frequency was also associated with larger Pe amplitude. These findings remained present after adjustment for confounding variables and were not moderated by sex.

**Conclusions:**

These findings show an important link between prevalent alcohol use behaviors and altered electrophysiological markers of error processing, representing a promising step forward in using large-scale electroencephalography for brain-alcohol use research and its clinical implications.

## Plain Language Summary

How well someone notices and reacts to their mistakes has been linked to their risk for alcohol addiction. We can measure how the brain processes mistakes with EEG (brain activity). In this study, we looked at brain responses in more than 1500 young people. We found that individuals who started drinking early or often drank a lot at once (called binge drinking) had different brain reactions to making mistakes. Studies such as this help us understand who may be at higher risk for alcohol addiction and how we can prevent it.

Alcohol use is often initiated during adolescence, a period that spans from approximately ages 10 through 20 to 24 years (1). During this developmental period, cortical regions that are involved in decision making and impulse control develop relatively slowly compared to the mesolimbic system, which is associated with reward processing (2, 3, 4, 5). This imbalance may contribute to increased risk-taking behaviors, including early alcohol use initiation (often defined as initiation before the age of 14/15 years) and binge drinking, which is often defined as consuming 4 (for women)/5 (for men) or more units of alcohol on one occasion (6, 7, 8). This can be concerning because early initiation has been linked to greater alcohol use later in life (9) and an increased risk of developing alcohol use disorder (AUD) (10, 11, 12). Furthermore, adolescent alcohol use has been associated with differences in brain morphology during adolescence and adulthood [for a review, see (13)].

While many large-scale epidemiological studies have focused on the interplay between brain morphology and alcohol use, less is known about the interplay between neurophysiological functioning and alcohol use in the general adolescent population. Electroencephalography (EEG) research has shown links between electrophysiological markers of attention allocation (e.g., the P3) and problematic alcohol use (14), as well as familial predisposition to AUD (15). Alcohol use and AUD have also been associated with electrophysiological markers of cognitive control (16), which is the ability to regulate thoughts and behaviors in accordance with goals and environmental demands (17). Within this framework, error processing, referring to the ability to detect mistakes and adjust behavior accordingly, may be particularly relevant. Theoretically, difficulty recognizing and learning from mistakes could increase the risk of exceeding intended alcohol use or escalating use over time, possibly leading to dependency symptoms. This is supported by several theoretical models (e.g., the dual-process models of addiction) which emphasize that the combination of decreased cognitive control and increased reward sensitivity forms a key risk factor for substance use (disorders) (18,19). Therefore, adolescence is seen as a critical window for either developing or preventing substance use problems.

Two commonly used EEG markers are the error-related negativity (ERN) and error-related positivity (Pe). After an erroneous response, a sharp negative peak (ERN) occurs in the EEG signal within around 100 ms (20), reflecting fast implicit error processing (21, 22, 23, 24), and is strongest over frontocentral electrode sites (e.g., FCz) (25). Later, around 200 to 500 ms after the erroneous response, a broader positive peak occurs (Pe), which is associated with conscious error awareness (26, 27, 28, 29, 30) and attentional processes that support behavioral adjustments (31). Additionally, time-frequency analyses can be used to uncover oscillatory components involved in error processing without the limitations of phase-locked averaging that is used in calculating event-related potentials (ERPs). Specifically, increased frontal midline theta (FM-theta) power (4–8 Hz) has been observed after erroneous responses in a time window overlapping with ERN/Pe latency (32, 33, 34, 35) and has been associated with behavioral measures of cognitive control (36, 37, 38).

These neurophysiological markers have been associated with varying degrees of substance use severity; meta-analyses and systematic reviews have shown associations between smaller ERN/Pe and substance use disorders (SUDs) in adults (39) and externalizing disorders (including addiction) (40) in children and adults, and a smaller ERN was even found in adult patients with SUDs compared to individuals with neurological disorders (41). In studies of alcohol use specifically, smaller ERN has been observed in heavy/binge young adult drinkers (42, 43, 44) and patients with AUD (45). However, a larger ERN has also been associated with binge drinking in young adults (46) and AUD in adults (47,48), and some studies have reported no association between ERN and heavy/binge drinking or only in subpopulations (49, 50, 51, 52). Although the relationship between Pe amplitude and alcohol use has been examined less often, there are indications that reduced Pe amplitude is related to heavy/binge drinking in emerging adults (51), indicating that adolescent alcohol use may also be related to conscious error processing that is not captured by the ERN. Reduced FM-theta power has been linked to alcohol use and AUD in multiple studies (53). Lower FM-theta power during a visual oddball task (a task to study attention allocation) has been prospectively associated with adolescent alcohol use in a large epidemiological sample (54), and adolescents with familial risk for AUD show decreased FM-theta power during a reward-processing task (55). A co-twin study suggests a potential causal relationship between alcohol use and reduced FM-theta power during NoGo trials, with stronger effects in women (56). In individuals with AUD, reduced FM-theta power has also been observed during NoGo trials (57). These findings highlight FM-theta power as both a potential risk marker and consequence of alcohol use, primarily in relation to broader cognitive control processes. This underscores the potential of FM-theta to capture additional aspects of error processing that are relevant to alcohol use and AUD risk.

Taken together, this literature seems to suggest that alcohol-related variations in neural markers of error processing are not only observed in (sub)clinical addiction populations but also in individuals who do not experience dependency symptoms. However, this is largely based on small-sample studies (20–50 per group) using convenience sampling (e.g., university students). Small samples often lack statistical power, which leads to inflated effects or a reduced ability to detect true associations, particularly when examining EEG-behavior associations (58, 59, 60). Moreover, even with a large sample size, findings from unrepresentative samples, which can be skewed by education, socioeconomic status, and sex distribution, may not reflect the broader population well.

Therefore, in the current study, we explored the relationship between EEG markers of error processing (i.e., ERN/Pe amplitude and FM-theta power) and alcohol use in a uniquely large adolescent sample (*N* = 1525) drawn from a population-based cohort. We accounted for potential confounding by adjusting models for sociodemographic factors, as well as alcohol use-related risk factors, going beyond the commonly used age and sex controls in EEG research. Finally, because studies suggest that reduced cognitive control is more pronounced in female heavy drinkers (42,50,61), we examined whether any associations were moderated by sex. Given the meta-analyses in patient studies, we hypothesized that smaller ERN and Pe amplitudes and (more exploratory) lower FM-theta power would be related to earlier alcohol use initiation, higher alcohol use quantity/frequency, and higher binge drinking frequency and that these associations would be stronger in female participants.

## Methods and Materials

### Study Population

**Figure 1:**
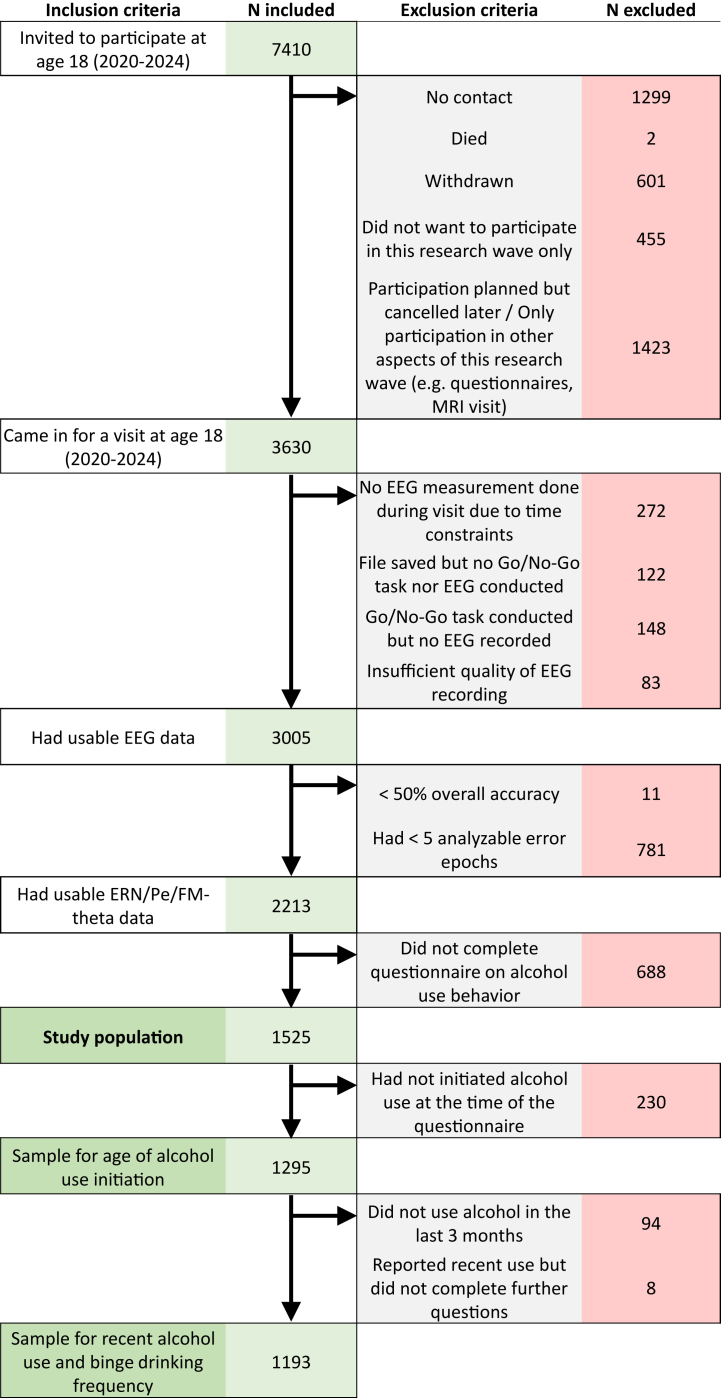
Flowchart of the analysis groups. EEG, electroencephalography; ERN, error-related negativity; FM-theta, frontal midline theta; MRI, magnetic resonance imaging; Pe, error-related positivity.

The Generation R Study is a population-based prospective cohort study from fetal life onwards that was designed to identify early environmental and genetic determinants of growth, development, and health. The cohort has been described in detail elsewhere (62). Briefly, pregnant women who were residents of Rotterdam, the Netherlands and whose delivery date was from April 2002 to January 2006 were invited to participate. Mother-child dyads were then invited for visits at the Erasmus Medical Center during multiple research waves (prenatal sample consisted of *N* = 9901 children). Participants age 17 to 18 years came in for the “Focus at 17” research visit at the Erasmus Medical Center (*n* = 3630). To avoid overburdening participants and considering the multiple assessments being performed during the visit, task duration and the number of electrodes used for EEG data collection was limited [see (63)]. The study population consisted of participants with a completed EEG measurement of sufficient quality, at least 50% task accuracy, a minimum of 5 analyzable error trials, and self-report of alcohol use behavior at around age 18 (*N* = 1525, see Figure 1 for a flowchart).

### EEG Acquisition

**Figure 2:**
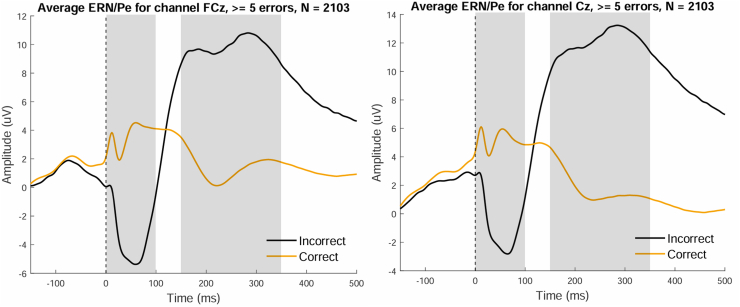
Grand average event-related potential waveforms recorded at electrode sites FCz and Cz. FCz and Cz averages are shown for participants with at least 5 analyzable error epochs. Response-locked to error responses, the waveforms show error-related negativity (ERN) and error-related positivity (Pe) components. Time windows for ERN and Pe are displayed in gray.

**Figure 3:**
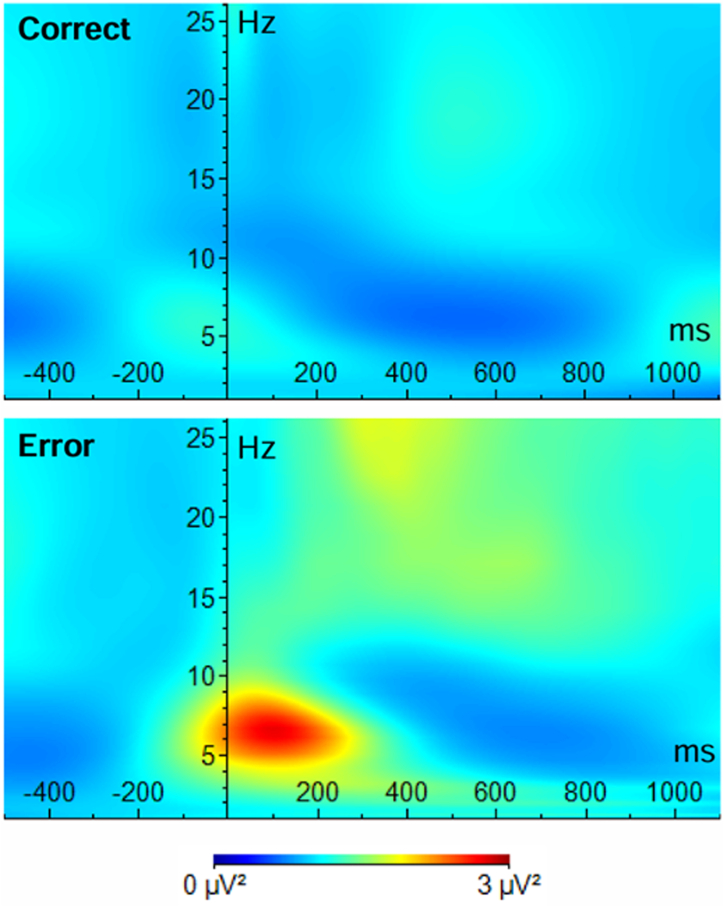
Time-frequency representations of power changes at electrode FCz for correct and error responses. Warmer colors indicate increased power relative to baseline, while cooler colors indicate decreased power. The 0 point indicates the time of response onset (correct/error). Theta-band (4–8 Hz) power increases can be observed after incorrect responses, while these are not present after correct responses.

EEG was recorded using a BioSemi Active-Two amplifier system in ActiView version 8.0 (BioSemi). The signal was recorded from 4 scalp sites (FCz, Cz, C3, and C4) with Ag/AgCl electrodes that were placed in an elastic cap. Because the ERPs we are interested in are maximum on midlines, and no hemispheric differences have been reported, only FCz and Cz electrodes were processed. Additionally, 4 electrodes were attached to left and right mastoids and infraorbital and supraorbital regions of the eye. Brain Vision Analyzer was used for data preprocessing. Detailed preprocessing steps for ERP and time-frequency analyses can be found in the Supplemental Methods. After preprocessing, data of *n* = 3005 participants were suitable for additional data analysis. Exclusion reasons included having Go/NoGo task data but no EEG data, having EEG data but only for a different task conducted during the same measurement, or having bad-quality EEG data (i.e., all error epochs contained an amplitude larger than ±100 μV, indicating noise). Grand average ERP waveforms (Figure 2) and time-frequency representations (Figure 3) were used to visualize Go/NoGo task-related neural dynamics. A minimum of 5 analyzable error epochs was chosen to balance EEG marker reliability and sample retention.

### Go/NoGo Task

The Go/NoGo task was used to assess error processing. Participants sat at a table with a button, resting their dominant index finger on it. They were shown stimuli (“W” or “M”) and were instructed to either press the button (Go for “W”) or withhold their response (NoGo for “M”). Participants were instructed to respond as quickly and accurately as possible, which requires both attentional control and inhibition of prepotent responses. Participants completed 160 randomized trials, 128 (80%) Go-trials and 32 (20%) NoGo trials. Each letter appeared for 800 ms or until a response was made, with intertrial intervals of 590 to 990 ms. Trials with reaction times below 200 ms were excluded as premature responses. Participants with accuracy below 50% were also excluded because this indicates difficulties understanding the task or lack of motivation to complete the task.

### Alcohol Use

Alcohol use information was derived from a questionnaire with various substance use-related questions. Alcohol use initiation was assessed with the question: “Have you ever drunk a whole alcoholic drink, or more than a few sips?” (yes/no). Age of alcohol initiation was assessed with the question “How old were you when you had your first alcoholic drink?,” with response options between “<10 years” and “≥17 years.” In analyses, response options “<10 years,” “10 years,” “11 years,” “12 years,” and “13 years” were combined into “≤13 years.” Alcohol use frequency/quantity in the last 3 months was assessed with the question: “How many units of alcohol did you drink on average in the last 3 months”? Response options included “<1 unit per week,” “1 to 3 units per week,” “4 to 6 units per week,” “1 unit per day,” “2 to 3 units per day,” and “>3 units per day.” The latter 3 response options were combined into “daily.” Binge drinking frequency was assessed with the question: “How often did you drink more than 4 units of alcohol in a single day in the last 3 months”? Response options were “never,” “once per month or less,” “more than monthly, less than weekly,” “once per week,” and “more than once per week.”

### Confounding Factors

Based on a previous study with the current dataset (63), we selected various variables as potential confounders for the relationship between EEG markers of error processing and adolescent alcohol use. A detailed description of the measures is provided in the Table S1. These variables were sex (male/female), IQ at around age 13 years (measured with a subset of the Wechsler Intelligence Scale for Children-Fifth Edition, i.e., vocabulary, matrix reasoning, digit span, and coding) (64), maternal education and household income around participant age 5 years, migration background, maternal self-reported use of alcohol and tobacco during pregnancy, and parental history of SUD (reported in prenatal questionnaires).

### Statistical Analyses

We calculated average ERP amplitudes (ERN/Pe), displayed grand average ERP waveforms (Figure 2), displayed grand-average time-frequency domains (Figure 3), and calculated average task behavior outcomes (i.e., mean reaction times and accuracy).

Then we used linear regression analyses to examine the associations between alcohol use variables and EEG markers of error processing (ERN and Pe mean amplitude and error-trial FM-theta power). Alcohol use variables included alcohol use initiation (yes/no), age at alcohol use initiation (in years), frequency/quantity of alcohol use in the last 3 months, and binge drinking frequency in the last 3 months. To assess whether associations followed a linear (ordinal) trend, we first conducted analyses using polynomial coding. Then we conducted pairwise comparisons between specific categories to identify where differences emerged. All analyses included unadjusted models (model 1); models adjusted for sex, IQ, maternal education, household income, and migration background (model 2); and models additionally adjusted for prenatal exposure to alcohol and tobacco and parental history of SUD (model 3). Second, we reran analyses with additional sex interactions. If a sex interaction was significant, simple slopes were calculated and plotted to determine the nature of the effect. Because our large sample size ensures validity under the central limit theorem, no corrections for nonnormality were performed.

We imputed missing data only on confounders (i.e., not on predictors or outcomes) using multiple imputation by chained equations (65), with 25 imputed datasets and 25 iterations per dataset. The percentage of missing data ranged from 0.7% for migration background to 42.5% for parental history of SUD, with most variables exhibiting around 10% to 20% missing values. All analyses were performed in R, version 4.2.1.

## Results

### Sample Description

In total, 2200 participants had EEG data of sufficient quality and reported on alcohol use around age 18 years. Of those, 1525 participants (806 female; 53%) had at least 5 analyzable error epochs, comprising the main analysis group [see (63) for the rationale behind this criterion]. Descriptive statistics for the sample (total and split by sex) are displayed in Tables 1 and 2. Of this analysis group, 1295 participants (84.9%) reported having initiated alcohol use, and 1193 (78.2%) reported alcohol use during the last 3 months. Standardized effect sizes for all main analyses are provided in Table 3, and statistical information can be found in the Supplemental Results and Table S2.

**Table 1:** Descriptive Statistics for the Study Sample (*N* = 1525), Based on the First Imputed Dataset

	Male, *n* = 719	Female, *n* = 806	Total, *N* = 1525
Age at EEG Measurement, Years	18.4 (0.6) [16.6–21.6]	18.4 (0.6) [16.5–21.4]	18.4 (0.6) [16.5–21.6]
IQ, WISC, Around 13 Years	103.0 (13.5) [62.0–140.0]	105.2 (12.5) [67.0–139.0]	104.1 (13.0) [62.0–140.0]
Migration Background			
Dutch background	515 (71.6%)	575 (71.3%)	1090 (71.5%)
Non-Western migration background	146 (20.3%)	165 (20.5%)	311 (20.4%)
Western migration background	58 (8.1%)	66 (8.2%)	124 (8.1%)
Maternal Education Level			
Low	54 (7.5%)	64 (7.9%)	118 (7.7%)
Mid-low	192 (26.7%)	223 (27.7%)	415 (27.2%)
Mid-high	216 (30.0%)	254 (31.5%)	470 (30.8%)
High	257 (35.7%)	265 (32.9%)	522 (34.2%)
Household Income per Month			
<€1600	62 (8.6%)	80 (9.9%)	142 (9.3%)
€1600–€4000	371 (51.6%)	406 (50.4%)	777 (51.0%)
>€4000	286 (39.8%)	320 (39.7%)	606 (39.7%)
Prenatal Exposure to Alcohol Use			
Mother never drank alcohol during pregnancy	227 (31.6%)	324 (40.2%)	551 (36.1%)
Mother drank alcohol until pregnancy was known	120 (16.7%)	106 (13.2%)	226 (14.8%)
Mother continued alcohol use during pregnancy	372 (51.7%)	376 (46.7%)	748 (49.0%)
Prenatal Exposure to Tobacco Use			
Mother never smoked during pregnancy	577 (80.3%)	619 (76.8%)	1196 (78.4%)
Mother smoked until pregnancy was known	52 (7.2%)	78 (9.7%)	130 (8.5%)
Mother continued smoking during pregnancy	90 (12.5%)	109 (13.5%)	199 (13.0%)
Parental History of Substance Use Disorder			
Yes	74 (10.3%)	102 (12.7%)	176 (11.5%)
No	645 (89.7%)	704 (87.3%)	1349 (88.5%)

A detailed description of the measures is provided in Table S1. Values are presented as mean (SD) [range] or *n* (%).

EEG, electroencephalography; WISC, Wechsler Intelligence Scale for Children-Fifth Edition.

**Table 2:** Descriptive Statistics for Electroencephalography and Alcohol Use Variables Used in the Current Study*,N* = 1525

	Male, *n* = 719	Female, *n* = 806	Total, *N* = 1525
FCz ERN Mean Amplitude, μV	−2.5 (7.7) [−28.3 to 25.0]	−3.8 (7.9) [−35.1 to 25.9]	−3.2 (7.9) [−35.1 to 25.9]
Cz PE Mean Amplitude, μV	12.6 (9.4) [−20.9 to 46.9]	12.5 (9.8) [−26.1 to 46.7]	12.5 (9.6) [−26.1 to 46.9]
FM-Theta Power, Error Trials, μV^2^	2.2 (0.8) [0.6–5.9]	2.3 (0.8) [0.7–6.1]	2.2 (0.8) [0.6–6.1]
NA^a^	0	1	1
Number of Analyzable Error Epochs	10.1 (4.1) [5.0–26.0]	9.3 (3.9) [5.0–26.0]	9.7 (4.0) [5.0–26.0]
Percentage Correct, %	90.1 (5.3) [55.0–96.9]	90.8 (4.7) [61.9–96.9]	90.4 (5.0) [55.0–96.9]
Alcohol Initiation, Yes/No			
No	107 (14.9%)	123 (15.3%)	230 (15.1%)
Yes	612 (85.1%)	683 (84.7%)	1295 (84.9%)
Age at Alcohol Initiation, Years			
NA^b^	107	123	230
Age 13 or younger	45 (7.4%)	67 (9.8%)	112 (8.6%)
Age 14	94 (15.4%)	125 (18.3%)	219 (16.9%)
Age 15	176 (28.8%)	213 (31.2%)	389 (30.0%)
Age 16	155 (25.3%)	155 (22.7%)	310 (23.9%)
Age 17 or older	142 (23.2%)	123 (18.0%)	265 (20.5%)
Any Alcohol Use During the Last 3 Months, Yes/No			
NA^b^	104	122	226
No	50 (8.1%)	49 (7.2%)	99 (7.6%)
Yes	565 (91.9%)	635 (92.8%)	1200 (92.4%)
Alcohol Use Quantity During the Last 3 Months			
NA^b^	153	176	329
<1 unit weekly	167 (29.5%)	232 (36.8%)	399 (33.4%)
1–3 units weekly	132 (23.3%)	162 (25.7%)	294 (24.6%)
4–6 units weekly	176 (31.1%)	175 (27.8%)	351 (29.3%)
Daily	91 (16.1%)	61 (9.7%)	152 (12.7%)
Binge Drinking Frequency During the Last 3 Months			
NA^b^	153	173	326
Never	101 (17.8%)	135 (21.3%)	236 (19.7%)
Once per month or less	130 (23.0%)	148 (23.4%)	278 (23.2%)
More than monthly, less than weekly	128 (22.6%)	191 (30.2%)	319 (26.6%)
Once per week	110 (19.4%)	87 (13.7%)	197 (16.4%)
More than once per week	97 (17.1%)	72 (11.4%)	169 (14.1%)

Values are presented as mean (SD) [range] or *n* (%).

ERN, error-related negativity; FM-theta, midfrontal theta power; NA, not available; Pe, error-related positivity.

a For 1 participant with data on ERN/Pe amplitudes, FM-theta power could not be calculated.

b These participants had missing data on these questions because they responded no to a previous question about their alcohol use (they either did not initiate alcohol use or did not report alcohol use in the last 3 months).

**Table 3:** Standardized Effect Sizes (Beta Coefficients) of Associations Between Alcohol Use Variables and Electroencephalography Markers of Error Processing

		ERN			Pe			FM-Theta (Error)		
		M1	M2	M3	M1	M2	M3	M1	M2	M3
Alcohol Use Initiation, *N* = 1525	–	0.011	0.004	0.005	0.053	0.034	0.033	−0.024	−0.036	−0.036
Alcohol Use Initiation Age, *n* = 1295 (Reference: Age 13 or Younger)	Age 14	−0.058	−0.056	−0.059	−0.020	−0.027	−0.027	0.048	0.042	0.040
	Age 15	−0.078^a^	−0.081^a^	−0.083^a^	−0.026	−0.032	−0.029	−0.027	−0.030	−0.032
	Age 16	−0.070	−0.071^a^	−0.070	−0.017	−0.016	−0.012	−0.003	0.000	−0.001
	Age 17 or older	−0.112^a^	−0.112^a^	−0.120^a^	−0.057	−0.050	−0.051	−0.049	−0.040	−0.042
Alcohol Use Quantity/Frequency, *n* = 1193 (Reference: <1 Unit Weekly)	1–3 units weekly	0.040	0.033	0.031	0.036	0.034	0.037	0.103^a^	0.106^a^	0.104^a^
	4–6 units weekly	0.050	0.037	0.037	0.068^a^	0.055	0.061	0.054	0.050	0.048
	Daily	0.012	−0.008	−0.012	0.072	0.047	0.050	0.061	0.047	0.042
Binge Drinking Frequency, *n* = 1193 (Reference: Never)	Once per month or less	0.043	0.047	0.049	0.068	0.063	0.064	−0.012	−0.014	−0.014
	More than monthly, less than weekly	0.051	0.056	0.056	0.070^a^	0.057	0.061	0.068	0.059	0.056
	Once per week	0.054	0.049	0.050	0.144^a^	0.124^a^	0.129^a^	0.009	−0.007	−0.010
	More than once per week	0.116^a^	0.109^a^	0.112^a^	0.141^a^	0.120^a^	0.125^a^	0.038	0.021	0.020

M1 was the unadjusted model; M2 was adjusted for sex, IQ, maternal education, household income, and migration background; and M3 was adjusted for all confounders of M2 and also for prenatal exposure to alcohol and tobacco and parental history of substance use disorder. Because ERN is a negatively deflected component, a lower value is equivalent to an absolute larger ERN amplitude, while a higher value is equivalent to an absolute smaller ERN amplitude.

ERN, error-related negativity; FM-theta (error), frontal midline theta power on error trials; Pe, error-related positivity.

a Significant pairwise comparisons (*p* < .05).

Notably, participants who made at least 5 analyzable errors during the NoGo task (and comprised the main analysis group) were more often male, had a lower IQ on average, and smaller Pe amplitudes and lower FM-theta power on error trials compared with participants who had between 1 and 4 analyzable error epochs (63). However, these groups did not differ on any alcohol use measure (initiation, initiation age, recent use, and recent binge drinking), ERN amplitude, or any of the other confounding variables (participant age, migration background, maternal education level, household income, prenatal alcohol/tobacco exposure, and family history of SUD).

### Alcohol Initiation

No association was observed between EEG markers of error processing (ERN, Pe, and FM-theta on error trials) and whether or not participants had initiated alcohol use at the time of self-report. This was true for unadjusted models and for the models that were adjusted for sociodemographic variables and alcohol-related risk factors. However, within the group that had initiated alcohol use (*n* = 1295), a significant linear trend was observed across alcohol use initiation age categories, such that earlier initiation age was associated with smaller absolute ERN amplitude. This was true for the unadjusted model, as well as for models adjusting for sociodemographic factors and models also adjusting for alcohol-related risk factors. Specifically, this association was observed when comparing the oldest initiators (who initiated at 17 years or older) to the youngest initiators (who initiated at 13 years or younger) and when comparing initiators at age 15 years to the youngest initiators (also see Table 3). A similar marginal association seemed to be present when we compared initiators at age 16 with the youngest initiators, but this only reached statistical significance in model 2 (adjusted for sociodemographic factors). Finally, a negative quartic trend was observed between FM-theta power and age of alcohol initiation in all models.

### Alcohol Use Frequency and Quantity, Including Binge Drinking

In a sample of participants who reported alcohol use during the last 3 months (*n* = 1193), we examined associations between EEG markers of error processing and frequency/quantity of alcohol use and frequency of binge drinking during the last 3 months. Importantly, a linear trend was observed across binge drinking categories: higher binge drinking frequency was associated with smaller absolute ERN amplitude after adjusting for sociodemographic factors and alcohol-related risk factors (also see Table 3). Furthermore, pairwise comparisons showed that participants who binge drank more than once per week showed absolute smaller ERN amplitudes than participants who reported binge drinking once a month or less. Surprisingly, we also observed a linear trend for Pe amplitudes: higher binge drinking was associated with larger Pe amplitude in all models. Pairwise comparisons showed that in unadjusted models, participants who either reported monthly binge drinking, weekly binge drinking, or binge drinking more than once a week all had larger Pe amplitudes than participants who reported no binge episodes in the last 3 months. When we adjusted for sociodemographic factors, as well as when we also adjusted for alcohol-related risk factors, participants who reported 1 or more binge drinking episodes per week showed a larger Pe amplitude than participants who reported no binge episodes in the last 3 months. Higher frequency/quantity of overall alcohol use was also associated with larger Pe amplitude in an unadjusted model: participants who consumed 4 to 6 units of alcohol a week showed larger Pe amplitude than participants who reported less than weekly alcohol use. However, this association did not remain after adjusting for confounding factors. Finally, a positive quartic trend was observed between FM-theta power and binge drinking frequency in all models.

### Sensitivity Analyses

As sensitivity analyses, we examined whether the relationship between EEG parameters and alcohol use (initiation, initiation age, recent alcohol quantity/frequency, and recent binge frequency) was moderated by sex. No significant sex interaction effects were observed.

## Discussion

In the current study, we examined the relationship between adolescent alcohol use and electrophysiological markers of error processing in a large sample drawn from a population-based cohort. Importantly, we observed associations between smaller absolute ERN amplitude, suggesting reduced implicit error processing, and variables associated with alcohol use disorder risk, i.e., early alcohol use initiation and high binge drinking frequency. Surprisingly, for higher binge drinking frequency specifically, we also observed an association with a larger Pe amplitude. These findings were not explained by factors such as sex, IQ, or socioeconomic factors or by alcohol-related risk factors such as prenatal alcohol and tobacco exposure or a parental history of SUD.

Our findings concerning the ERN are consistent with literature reporting a relationship between reduced ERN amplitude and heavy or binge drinking in young adults (42, 43, 44) and AUD in adults (45), reflecting the theoretical link between decreased cognitive control and SUD risk (18,19). Furthermore, this is the first study to show a link between reduced ERN and early initiation of alcohol use; specifically, initiation at age 13 or younger compared to initiation at age 15 or older. Early alcohol use initiation is an important predictor of SUD later in life [for a review, see (66)], and there are indications that this is largely explained by common genetic risk factors (67). However, in the current study, the association between ERN and early initiation remained after controlling for parental history of SUD. This suggests that reduced ERN may be a specific marker for early initiation in addition to an alcohol use vulnerability linked to familial risk, which the ERN has also been associated with (68,69). However, it should be taken into account that parental SUD was assessed prenatally and does not reflect a parental SUD that developed during the participant’s upbringing. Alternatively, the reduced ERN could reflect a neuromodulatory effect of prolonged alcohol exposure resulting from early initiation, speculating based on findings that acute alcohol intoxication decreases ERN in healthy individuals (70,71). At the same time, our ERN findings are inconsistent with research reporting larger ERN in youth who binge drink (46) and patients with AUD (47,48) and with null findings on the ERN-alcohol association (49, 50, 51, 52). However, the larger ERN in the literature (47,48) may partially reflect anxiety and withdrawal symptoms (72, 73, 74, 75, 76) and/or small samples and specific group composition (46).

Surprisingly, we observed a robust association between larger Pe and frequent binge drinking (at least weekly), suggesting increased conscious error processing. This was not explained by IQ or maternal education, which were associated with larger Pe in our previous study using a larger sample of the same cohort (63), or larger NoGo P3 in children (77) and adolescents (63,78). Because the link between Pe and binge drinking has not been explored much, and existing studies have reported either a reduced (51) or delayed Pe (46), the nature of this finding remains unclear. It should be noted that Pe was measured at Cz and not at CPz or Pz, which are also commonly used Pe sites. Although a significant association at Cz likely reflects a similar association at more posterior locations, we cannot rule out that measuring at CPz or Pz might have yielded different results.

Nevertheless, our findings indicate, together with previous research, that ERN and Pe reflect distinct processes with separate functions rather than largely overlapping aspects of error processing. The distinction between the motivational aspect of the Pe (79) and the implicit error processing of the ERN (26,80) might be more pronounced for certain behavioral patterns such as binge drinking. Alternatively, a compensatory hypothesis has been proposed in EEG studies reporting larger N2 and P3 in binge drinkers during a Flanker task (50) and tasks assessing attention allocation (81,82) and working memory (83,84). When applied to our findings, this hypothesis suggests that regular binge drinkers recruit additional cognitive resources (reflected by larger Pe) to maintain task performance, potentially compensating for reduced implicit error processing (reflected by smaller ERN).

Finally, we observed a complex nonlinear relationship between FM-theta power and alcohol use variables, which warrants additional investigation. While adolescent EEG studies linking alcohol use and AUD to lower FM-theta power exist [for a review, see (53, 54, 55, 56, 57)], their focus was not on error-related FM-theta. Therefore, more research is needed to clarify the robustness and nature of this association.

A key strength of this study is its uniquely large sample size, leveraging the reliability of EEG markers (58) and providing sufficient variability in alcohol use measures (112–1295 participants per category). This study is also one of the first to examine EEG markers of cognitive control and alcohol use in a diverse sample drawn from a population-based cohort, increasing generalizability of the current findings. Regardless, further studies should aim to replicate these findings using more heterogenous samples across various sociocultural contexts to improve generalizability. Furthermore, decades of data were leveraged to adjust for confounders like IQ, socioeconomic factors, sex, and alcohol-related risk factors, strengthening the validity of reported EEG-alcohol use links. However, as these links are cross-sectional and subject to potential residual confounding, no causal relationship between EEG markers and alcohol use can be assumed. Thus, the current study is unable to address the question whether variations in EEG measures form a liability for or a consequence of adolescent alcohol use. Another important limitation is the short EEG measurement duration, which necessitated a relatively low minimum error threshold to retain enough participants and minimize selection bias, but still resulted in substantial exclusions, potentially introducing selection bias. Finally, alcohol use was assessed with non-standardized questions, limiting comparability with existing and future research.

### Conclusions

In conclusion, the current study indicates robust associations between electrophysiological markers of error processing and AUD-related risk factors such as early initiation and frequent binge drinking in a large diverse sample. This marks an important step toward employing large-scale EEG to study brain-behavior relationships, which has been considered highly promising for clinical impact due to its scalability and cost-effectiveness (85, 86, 87). Longitudinal studies are needed to examine directionality (i.e., whether EEG variations precede or result from alcohol use) and underlying mechanisms of the EEG-alcohol use association. Furthermore, any translation to individual-level clinical utility may depend on the application of multivariate and machine learning approaches and the use of predictive methods (88). Together with the current findings, these research efforts could have an important contribution to early detection, prevention, and targeted interventions for individuals at risk of developing AUD.

## Supplementary Material

Supplemental Methods, Results, and Table S1

Table S2

Key Resources Table
