# Supplementary material for: Adolescent Risky Alcohol Use Is Associated With Electrophysiological Markers of Error Processing: Findings From a Large Cohort Study
Source: Biol Psychiatry Glob Open Sci. 2025 Sep 17;6(1):100615. doi: 10.1016/j.bpsgos.2025.100615 (PMC12593575; doi:10.1016/j.bpsgos.2025.100615)
Supplement: Supplemental Methods, Results, and Table S1 [file mmc1.pdf]

SUPPLEMENTARY INFORMATION

Adolescent Risky Alcohol Use Is Associated With Electrophysiological Markers of Error Processing: Findings from a Large Cohort Study

Boer *et al.*

Supplemental Methods: page 2

Supplemental Results: page 3

Supplemental Table 1: page 6

References: page 9

Supplemental Methods

EEG acquisition

EEG was recorded using a BioSemi Active-Two amplifier system in ActiView version 8.0 (BioSemi, Amsterdam, the Netherlands). The signal was recorded from 4 scalp sites (FCz, Cz, C3 and C4) with Ag/AgCl electrodes that were placed in an elastic cap. Since the ERPs we are interested in are maximum on midlines and no hemispheric differences have been reported, only FCz and Cz electrodes were processed. Additionally, four electrodes were attached to left and right mastoids, and infraorbital and supraorbital regions of the eye (VEOG). Brain Vision Analyzer was used for data preprocessing. Offline, the EEG signal was re-referenced to computed linked mastoids. EEG and EOG activity were filtered with a bandpass of .1–35 Hz (phase shift-free Butterworth filters; 24 dB/octave slope). Then, the data were segmented in epochs. For the error processing analyses (ERN/Pe), we used a time window of 1100 ms; 300 ms before until 800 ms after a response. Next, an ocular correction was applied on all data (Gratton et al., 1983). The segment of 300 to 100 ms before the response was used for baseline correction. Finally, epochs that contained an amplitude larger than +/-100 µV (indicative of noise) were rejected. For participants where 50% or more of the epochs were rejected, the EEG data was visually inspected to determine whether the issue was caused by one of the mastoid references distorting the signal. In this case, these participants' data were re-referenced to only the properly functioning mastoid reference.

After preprocessing, data of N = 3005 participants was suitable for further data analysis. Exclusion reasons included having Go/No-Go task data but no EEG data, having EEG data but only for a different task conducted during the same measurement, or having bad quality EEG data. ERN and Pe amplitude were defined as the mean value (in µV) in the 0-100 ms and 150-350 ms time segments following an incorrect response, respectively.

For ERN, we focused on electrode FCz (Bush et al., 2000; Van Veen & Carter, 2002; Yeung et al., 2004). The Pe is more widely distributed, and shows up initially frontocentral but shifting to centroparietal regions between 300–500 ms post-error (Ullsperger et al., 2014; Van Veen & Carter, 2002). It has been reported as maximal at FCz, Cz, CPz, or Pz depending on the time window, with Cz and CPz often showing the largest amplitudes (Endrass et al., 2012; Ridderinkhof et al., 2009). In the present study, we focused on Cz.

ERN/Pe parameters were based on a mean number of 7.8 (SD = 4.6, range 1-26) analyzable error-epochs in the full sample. In the subsample for the ERN/Pe Cronbach's alpha calculation, in which

participants with at least 10 error epochs were selected, ERN/Pe parameters were based on a mean number of 13.3 (SD = 3.2, range 10-26) analyzable error-epochs.

For the time-frequency analysis, EEG activity was first filtered with a bandpass of 1-40 Hz (phase shift-free Butterworth filters; 24 dB/octave slope). Then, the data were segmented into epochs of 3000 ms, starting from 1500 ms prior to stimulus/response onset to 1500 ms following stimulus/response onset. Next, ocular correction was performed (Gratton et al., 1998). For baseline correction, a time window of 1500 to 0 ms before a stimulus/response was used. Epochs that contained an amplitude larger than +/-100 µV (indicative of noise) were rejected. Data were segmented separately per trial type (i.e., Go, No-Go, correct, error). The time-frequency analysis was applied using continuous wavelet transformation (Morlet complex waveform, frequency range from 1 to 40 Hz in 40 logarithmic steps, Morlet parameter c = 7). Then, the mean amplitude values and percent change were calculated per trial type, with a reference time of -400 ms to -100 ms. Following, a wavelet data export was applied for each trial type in order to calculate theta power using Data Normalization Type with Point Mean. Here, theta was quantified within the range of 4.13 to 8.01 Hz (using layers 16 to 23), and measured at electrode site FCz, as is recommended in the literature (Cavanagh & Frank, 2014; Cohen, 2011; Fusco et al., 2018; Zheng & Wynn, 2022). For correct and error trials, a time window of 0 to 200 ms was used for FM-theta power calculations.

Supplemental Results (Original text, including statistical information)

Alcohol initiation

No association was observed between EEG markers of error processing (ERN, Pe and FM-theta on error trials) and whether or not participants had initiated alcohol use at the time of self-report. This was the case for unadjusted models, and for the models that were adjusted for sociodemographic variables and alcohol-related risk factors. However, within the group that had initiated alcohol use (N = 1295), a significant linear trend was observed across alcohol use initiation age categories, such that earlier initiation age was associated with smaller absolute ERN amplitude. This was the case for the unadjusted model (b = -1.80, SE = .60, t(1287.88) = -2.99, p = .003), as well as models adjusting for sociodemographic factors (b = -1.83, SE = .60, t(1275.28) = -3.01, p = .003), and models additionally adjusting for alcohol-related risk factors (b = -1.90, SE = .61, t(1264.55) = -3.11, p = .002). Specifically, this association was observed when comparing the oldest initiators (who initiated at 17 years or older)

to the youngest initiators (who initiated at 13 years or younger) (**M1**: *b* = -2.70, *SE* = .89, *t*(1287.88) = -3.04, *p* = .002; **M2**: *b* = -2.70, *SE* = .89, *t*(1275.16) = -3.04, *p* = .002; **M3**: *b* = -2.88, *SE* = .90, *t*(1265.65) = -3.20, *p* = .001), and when comparing initiators at age 15 years to the youngest initiators (**M1**: *b* = -1.87, *SE* = .84, t(1287.88) = -2.22, *p* = .03; **M2**: *b* = -1.96, *SE* = .84, *t*(1277.19) = -2.32, *p* = .02; **M3**: *b* = -1.99, *SE* = .85, *t*(1262.67) = -2.34, *p* = .02). A similar marginal association seemed to be present when comparing initiators at age 16 with the youngest initiators, but this only reached statistical significance in Model 2 (*b* = -1.71, *SE* = .87, *t*(1276.48) = -1.97, *p* = .049). Finally, a negative quartic trend was observed between FM-theta power and age of alcohol initiation in all models.

Alcohol use frequency and quantity, including binge drinking

In a sample of participants reporting alcohol use in the last three months (N = 1193), we examined associations between EEG markers of error processing and frequency/quantity of alcohol use, and frequency of binge drinking in the last three months. Importantly, a linear trend was observed across binge drinking categories: higher binge drinking frequency was associated with smaller absolute ERN amplitude, also after adjusting for sociodemographic factors and alcohol-related risk factors (also see **Table 3**) (**M1**: *b* = 1.49, *SE* = .56, *t*(1185.89) = 2.67, *p* = .008; **M2**: *b* = 1.35, *SE* = .58, *t*(1174.64) = 2.32, *p* = .02; **M3**: *b* = 1.37, *SE* = .58, *t*(1160.42) = 2.35, *p* = .02). Furthermore, pairwise comparisons showed that participants who binge drank more than once per week showed absolute smaller ERN amplitudes as compared to participants who reported to binge once a month or less (**M1**: *b* = 2.24, *SE* = .80, *t*(1185.89) = 2.80, *p* = .005; **M2**: *b* = 2.11, *SE* = .82, *t*(1174.66) = 2.57, *p* = .01; **M3**: *b* = 2.16, *SE* = .82, *t*(1165.27) = 2.62, *p* = .01). Surprisingly, we also observed a linear trend for Pe amplitudes: higher binge drinking was associated with larger Pe amplitude in all models (**M1**: *b* = 2.69, *SE* = .68, *t*(1185.89) = 3.96, *p* < .001; **M2**: *b* = 2.26, *SE* = .71, *t*(1173.00) = 3.19, *p* = .001; **M3**: *b* = 2.36, *SE* = .72, *t*(1158.70) = 3.30, *p* = .001). Pairwise comparisons showed that in unadjusted models, participants who either reported monthly binge drinking (*b* = 1.66, *SE* = .83, *t*(1185.89) = 1.99, *p* = .047), weekly binge drinking (*b* = 3.43, *SE* = .94, *t*(1185.89) = 3.66, *p* < .001) or binge drinking more than once a week (*b* = 3.35, *SE* = .98, *t*(1185.89) = 3.43, *p* < .001), all had larger Pe amplitudes as compared to participants who reported no binge episodes in the last three months. When adjusting for sociodemographic factors, as well as when additionally adjusting for alcohol-related risk factors, participants who reported one (**M2:** *b* = 2.94, *SE* = .96, *t*(1174.24) = 3.06, *p* = .002; **M3**: *b* = 3.07, *SE* = .97, *t*(1159.81) = 3.16, *p* = .002) or more (**M2**: *b* = 2.85, *SE* = 1.01, *t*(1174.08) = 2.84, *p* = .005; **M3**: *b* = 2.96, *SE* = 1.01, *t*(1163.50) = 2.93, *p* = .003) binge drinking

episodes per week showed a larger Pe amplitude as compared to participants reporting no binge episodes in the last three months. Higher frequency/quantity of overall alcohol use was also associated with larger Pe amplitude in an unadjusted model: participants who consumed 4-6 units of alcohol a week showed larger Pe amplitude as compared to participants who reported less than weekly alcohol use (b = 1.47, SE = .71, t(1186.89) = 2.07, p = .04). However, this association did not remain after adjusting for confounding factors. Finally, a positive quartic trend was observed between FM-theta power and binge drinking frequency in all models.

Sensitivity analyses

As sensitivity analyses, we examined whether the relationship between EEG parameters and alcohol use (initiation, initiation age, recent alcohol quantity/frequency and recent binge frequency) was moderated by sex. No significant sex interaction effects were observed.

Supplemental Table 1. *Confounding factors in this study, how they were operationalized and literature on the association of the confounding factors with our variables of interest (alcohol use and EEG parameters).*

Variable	Operationalization	Literature on association with 1) alcohol use and 2) EEG parameters
**Child sex**	Biological sex assigned at birth (male/female) was used in this study because the sample is drawn from a prospective birth cohort, and gender identity, which can develop and be expressed later in life, could not be assessed at the time of birth. Therefore, sex assigned at birth serves as the most consistent and available variable for analyses within this cohort.	1)<br>McHugh et al. (2018)<br>Raninen et al. (2024)<br><br>2)<br>Boer et al. (2025 [Preprint])<br>Fischer et al. (2016)<br>Imburgio et al. (2020)<br>Larson et al. (2011)<br>Strand et al. (2021)
**Child IQ around age 13**	A subset of the Wechsler Intelligence Scale for Children-Fifth Edition (WISC-V) was used (Wechsler, 2014). The WISC-V is an instrument assessing individual cognitive functioning in 6 to 16-year-olds. In collaboration with Pearson (Pearson Clinical Assessment, San Antonio, TX, US), four core subtests from the WISC-V were selected to assess specific cognitive domains and to derive an estimated FSIQ: Vocabulary, matrix reasoning, digit span and coding.	1)<br>Sjolund et al. (2012)<br>Sjolund et al. (2015)<br><br>2)<br>Boer et al. (2025 [Preprint])<br>Zijlmans et al. (2019)<br>Danovitch et al. (2019)<br>Steele et al. (2015)
**Child migration background**	Migration background was defined according to the classification of Statistics Netherlands (Alders, 2001): (1) if both parents are born in the Netherlands, there is no migration background and children are labeled Dutch; (2) if one of the parents is born in another country than the Netherlands, that country counts; (3) if both parents are born in the same country other than the Netherlands, that country counts; (4) if the parents are born in the different countries other than the Netherlands, the country of mothers counts; and (5) if that person and both parents are born in	1)<br>Chartier and Caetano (2010)<br>Trimbos-instituut (2025)<br><br>2)<br>Boer et al. (2025 [Preprint])

	different countries other than the Netherlands, the country of birth of that specific person counts. Originally categorized into Dutch, Indonesian, Cape Verdean, Moroccan, Dutch Antilles, Surinamese, Turkish, European, Asian and Other (African, American and Oceanian). Recoded into Dutch, non-Dutch western (European, American western, Asian western, Oceania), and non-Dutch non-western (Moroccan, Turkish, African, Dutch Antilles, Cape Verdean, Surinamese, Indonesian, American non-western, Asian non-western).	
**Maternal education at child age 5**	Highest attained educational level, categorized as:<br>Low (primary school; lower vocational training; intermediate general school; 3 years general secondary school), which typically corresponds to ≤12 years of education; Mid-low (>3 years general secondary school; intermediate vocational training; 1st year higher vocational training), in general corresponding with 13–15 years of education; Mid-high (higher vocational training; Bachelor's degree), typically matching with 16 or 17 years of education; and High (higher academic education; PhD), usually indicating 18 years of education or more (Statistics Netherlands: *Standard classification of education 2003 (Standaard onderwijsindeling 2003)*, 2004).	1)<br>Tolstrup et al. (2023)<br>Collins (2016)<br><br>2)<br>Boer et al. (2025 [Preprint])<br>Conejero et al. (2018)<br>Brooker (2018)<br>Isbell et al. (2024)
**Net household income at child age 5**	Categorized as: Less than €1600/month; Between €1600 and 4000/month; and more than €4000/month.	1)<br>Tolstrup et al. (2023)<br>Collins (2016)<br><br>2)<br>Boer et al. (2025 [Preprint])<br>Conejero et al. (2018)<br>Brooker (2018)<br>Isbell et al. (2024)

**Prenatal alcohol exposure**	Maternal alcohol use during pregnancy was categorized into 'never drank in pregnancy', 'drank until pregnancy was known', and 'continued to drink during pregnancy'. Notably, women who reported any alcohol use in the second or third trimester, were categorized into 'continued to drink during pregnancy'.	1)<br>Duko, Pereira, Tait, Bedaso, et al. (2022)<br><br>2)<br>Pini et al. (2024)
**Prenatal tobacco exposure**	Maternal tobacco smoking during pregnancy was categorized into 'never smoked during pregnancy', 'smoked until pregnancy was known', and 'continued smoking during pregnancy'.<br>Notably, women who reported any tobacco use in the second or third trimester, were categorized into 'continued smoking during pregnancy'.	1)<br>Duko, Pereira, Tait, Betts, et al. (2022)<br>Hunnicutt-Ferguson et al. (2025)<br><br>2)<br>Pini et al. (2024)
**Parental history of substance use disorder**	Based on the question "Have you ever been addicted to one or more substances?". Original options included 'Yes', 'Probably', 'Probably not', 'No' and 'Don't know' for both parents, and were recoded as follows: 'Yes' and 'Probably' were recoded into 'Yes'; 'No', 'Probably not' and 'Don't know' were recoded into 'No'. Maternal and paternal variables were combined into one variable as follows: if at least one of the parents had 'Yes', parental history of SUD was coded as 'Yes', otherwise it was coded as 'No'. Reported during the prenatal research phase.	1)<br>Verhulst et al. (2015)<br><br>2)<br>Euser et al. (2013)<br>Fein and Chang (2008)

References

Alders, M. (2001). Classification of the population with a foreign background in the Netherlands. Statistic Netherlands, paper for the conference „The measure and mismeasure of populations. The statistical use of ethnic and racial categories in mul ticultural societies", paris,

Boer, O. D., Lutz, M. C., El Marroun, H., Hajcak, G., & Franken, I. H. A. (2025). Reliability of Electrophysiological Measures of Cognitive Control and Sociodemographic Correlates in a Large Adolescent Cohort. https://doi.org/https://doi.org/10.31234/osf.io/3wabk_v1

Brooker, R. J. (2018). Maternal Behavior and Socioeconomic Status Predict Longitudinal Changes in Error-Related Negativity in Preschoolers. *Child Dev*, *89*(3), 725-733. https://doi.org/10.1111/cdev.13066

Bush, G., Luu, P., & Posner, M. I. (2000). Cognitive and emotional influences in anterior cingulate cortex. *Trends Cogn Sci*, *4*(6), 215-222. https://doi.org/S1364-6613(00)01483-2 [pii] 10.1016/s1364-6613(00)01483-2

Cavanagh, J. F., & Frank, M. J. (2014). Frontal theta as a mechanism for cognitive control. *Trends Cogn Sci*, *18*(8), 414-421. https://doi.org/S1364-6613(14)00107-7 [pii] 10.1016/j.tics.2014.04.012

Chartier, K., & Caetano, R. (2010). Ethnicity and health disparities in alcohol research. *Alcohol Res Health*, *33*(1-2), 152-160. https://doi.org/arh-33-1_2-152 [pii]

Cohen, M. X. (2011). Error-related medial frontal theta activity predicts cingulate-related structural connectivity. *Neuroimage*, *55*(3), 1373-1383. https://doi.org/S1053-8119(10)01675-7 [pii] 10.1016/j.neuroimage.2010.12.072

Collins, S. E. (2016). Associations Between Socioeconomic Factors and Alcohol Outcomes. *Alcohol Res*, *38*(1), 83-94. https://doi.org/arcr-38-1-83 [pii]

Conejero, A., Guerra, S., Abundis-Gutierrez, A., & Rueda, M. R. (2018). Frontal theta activation associated with error detection in toddlers: influence of familial socioeconomic status. *Dev Sci*, *21*(1). https://doi.org/10.1111/desc.12494

Danovitch, J. H., Fisher, M., Schroder, H., Hambrick, D. Z., & Moser, J. (2019). Intelligence and Neurophysiological Markers of Error Monitoring Relate to Children's Intellectual Humility. *Child Dev*, *90*(3), 924-939. https://doi.org/10.1111/cdev.12960

Duko, B., Pereira, G., Tait, R. J., Bedaso, A., Newnham, J., Betts, K., & Alati, R. (2022). Prenatal alcohol exposure and offspring subsequent alcohol use: A systematic review. *Drug Alcohol Depend*, *232*, 109324. https://doi.org/S0376-8716(22)00061-8 [pii] 10.1016/j.drugalcdep.2022.109324

Duko, B., Pereira, G., Tait, R. J., Betts, K., Newnham, J., & Alati, R. (2022). Prenatal alcohol and tobacco exposures and the risk of cannabis use in offspring: Findings from a population-based cohort study. *Neurotoxicol Teratol*, *90*, 107064. https://doi.org/S0892-0362(22)00002-2 [pii] 10.1016/j.ntt.2022.107064

Endrass, T., Klawohn, J., Preuss, J., & Kathmann, N. (2012). Temporospatial dissociation of Pe subcomponents for perceived and unperceived errors. *Front Hum Neurosci*, *6*, 178. https://doi.org/10.3389/fnhum.2012.00178

Euser, A. S., Evans, B. E., Greaves-Lord, K., Huizink, A. C., & Franken, I. H. (2013). Diminished error-related brain activity as a promising endophenotype for substance-use disorders: evidence from high-risk offspring. *Addict Biol*, *18*(6), 970-984. https://doi.org/10.1111/adb.12002

Fein, G., & Chang, M. (2008). Smaller feedback ERN amplitudes during the BART are associated with a greater family history density of alcohol problems in treatment-naive alcoholics. *Drug Alcohol*

Depend, *92*(1-3), 141-148. https://doi.org/S0376-8716(07)00282-7 [pii] 10.1016/j.drugalcdep.2007.07.017

Fischer, A. G., Danielmeier, C., Villringer, A., Klein, T. A., & Ullsperger, M. (2016). Gender Influences on Brain Responses to Errors and Post-Error Adjustments. *Sci Rep*, *6*, 24435. https://doi.org/srep24435 [pii] 10.1038/srep24435

Fusco, G., Scandola, M., Feurra, M., Pavone, E. F., Rossi, S., & Aglioti, S. M. (2018). Midfrontal theta transcranial alternating current stimulation modulates behavioural adjustment after error execution. *Eur J Neurosci*, *48*(10), 3159-3170. https://doi.org/10.1111/ejn.14174

Gratton, G., Coles, M. G., & Donchin, E. (1983). A new method for off-line removal of ocular artifact. *Electroencephalogr Clin Neurophysiol*, *55*(4), 468-484. https://doi.org/0013-4694(83)90135-9 [pii] 10.1016/0013-4694(83)90135-9

Hunnicutt-Ferguson, K., Stoner, S. A., Kable, J. A., Grant, T. M., & Coles, C. D. (2025). Substance use and mental health symptoms in adults with prenatal alcohol exposure. *Neurotoxicol Teratol*, *109*, 107436. https://doi.org/S0892-0362(25)00013-3 [pii] 10.1016/j.ntt.2025.107436

Imburgio, M. J., Banica, I., Hill, K. E., Weinberg, A., Foti, D., & MacNamara, A. (2020). Establishing norms for error-related brain activity during the arrow Flanker task among young adults. *Neuroimage*, *213*, 116694. https://doi.org/S1053-8119(20)30181-6 [pii] 10.1016/j.neuroimage.2020.116694

Isbell, E., Rodas De Leon, N. E., & Richardson, D. M. (2024). Childhood family socioeconomic status is linked to adult brain electrophysiology. *PLoS One*, *19*(8), e0307406. https://doi.org/PONE-D-24-06503 [pii] 10.1371/journal.pone.0307406

Larson, M. J., South, M., & Clayson, P. E. (2011). Sex differences in error-related performance monitoring. *Neuroreport*, *22*(1), 44-48. https://doi.org/00001756-201101050-00010 [pii] 10.1097/WNR.0b013e3283427403

McHugh, R. K., Votaw, V. R., Sugarman, D. E., & Greenfield, S. F. (2018). Sex and gender differences in substance use disorders. *Clin Psychol Rev*, *66*, 12-23. https://doi.org/S0272-7358(17)30250-7 [pii] 10.1016/j.cpr.2017.10.012

Pini, N., Sania, A., Rao, S., Shuffrey, L. C., Nugent, J. D., Lucchini, M., McSweeney, M., Hockett, C., Morales, S., Yoder, L., Ziegler, K., Perzanowski, M. S., Fox, N. A., Elliott, A. J., Myers, M. M., & Fifer, W. P. (2024). In Utero Exposure to Alcohol and Tobacco and Electroencephalogram Power During Childhood. *JAMA Netw Open*, *7*(1), e2350528. https://doi.org/2813608 [pii] zoi231476 [pii] 10.1001/jamanetworkopen.2023.50528

Raninen, J., Ramstedt, M., Thor, S., & Torronen, J. (2024). Mind the gap! Gender differences in alcohol consumption among Swedish ninth graders 1989-2021. *Drug Alcohol Rev*, *43*(3), 596-603. https://doi.org/10.1111/dar.13718

Ridderinkhof, K. R., Ramautar, J. R., & Wijnen, J. G. (2009). To P(E) or not to P(E): a P3-like ERP component reflecting the processing of response errors. *Psychophysiology*, *46*(3), 531-538. https://doi.org/10.1111/j.1469-8986.2009.00790.x

Sjolund, S., Allebeck, P., & Hemmingsson, T. (2012). Intelligence quotient (IQ) in adolescence and later risk of alcohol-related hospital admissions and deaths--37-year follow-up of Swedish conscripts. *Addiction*, *107*(1), 89-97. https://doi.org/10.1111/j.1360-0443.2011.03544.x

Sjolund, S., Hemmingsson, T., & Allebeck, P. (2015). IQ and level of alcohol consumption-findings from a national survey of Swedish conscripts. *Alcohol Clin Exp Res*, *39*(3), 548-555. https://doi.org/10.1111/acer.12656

Standard classification of education 2003 (Standaard onderwijsindeling 2003). (2004). http://www.cbs.nl

Steele, V. R., Claus, E. D., Aharoni, E., Vincent, G. M., Calhoun, V. D., & Kiehl, K. A. (2015). Multimodal imaging measures predict rearrest. *Front Hum Neurosci*, *9*, 425. https://doi.org/10.3389/fnhum.2015.00425

Strand, N., Fang, L., & Carlson, J. M. (2021). Sex Differences in Anxiety: An Investigation of the Moderating Role of Sex in Performance Monitoring and Attentional Bias to Threat in High Trait Anxious Individuals. *Front Hum Neurosci*, *15*, 627589. https://doi.org/10.3389/fnhum.2021.627589

Tolstrup, J. S., Kruckow, S., Becker, U., Andersen, O., Sawyer, S. M., Katikireddi, S. V., & Moller, S. P. (2023). Socioeconomic inequalities in alcohol-related harm in adolescents: a prospective cohort study of 68,299 Danish 15-19-year-olds. *EClinicalMedicine*, *62*, 102129. https://doi.org/S2589-5370(23)00306-1 [pii] 102129 [pii] 10.1016/j.eclinm.2023.102129

Trimbos-instituut. (2025). *Nationale Drug Monitor, editie 2025.* Trimbos-instituut, Utrecht & WODC, Den Haag. Retrieved April 7th from https://www.nationaledrugmonitor.nl/

Ullsperger, M., Danielmeier, C., & Jocham, G. (2014). Neurophysiology of performance monitoring and adaptive behavior. *Physiol Rev*, *94*(1), 35-79. https://doi.org/94/1/35 [pii] 10.1152/physrev.00041.2012

Van Veen, V., & Carter, C. S. (2002). The timing of action-monitoring processes in the anterior cingulate cortex. *J Cogn Neurosci*, *14*(4), 593-602. https://doi.org/10.1162/08989290260045837

Verhulst, B., Neale, M. C., & Kendler, K. S. (2015). The heritability of alcohol use disorders: a meta-analysis of twin and adoption studies. *Psychol Med*, *45*(5), 1061-1072. https://doi.org/S0033291714002165 [pii] 10.1017/S0033291714002165

Wechsler, D. (2014). *WISC-V: Technical and interpretive manual*. NCS Pearson, Incorporated.

Yeung, N., Botvinick, M. M., & Cohen, J. D. (2004). The neural basis of error detection: conflict monitoring and the error-related negativity. *Psychol Rev*, *111*(4), 931-959. https://doi.org/2004-19012-005 [pii] 10.1037/0033-295x.111.4.939

Zheng, X. Y., & Wynn, S. C. (2022). Midfrontal theta is associated with errors, but no evidence for a link with error-related memory. *Neuroimage: Reports*, *2*(4), 100129. https://doi.org/https://doi.org/10.1016/j.ynirp.2022.100129

Zijlmans, J., Bevaart, F., van Duin, L., Luijks, M. J. A., Popma, A., & Marhe, R. (2019). Error-related brain activity in relation to psychopathic traits in multi-problem young adults: An ERP study. *Biol Psychol*, *144*, 46-53. https://doi.org/S0301-0511(18)30508-8 [pii] 10.1016/j.biopsycho.2019.03.014
